# Single-Cell and Mendelian Randomization Analyses Reveal Novel Genetic Risk Genes of Perivascular Macrophages in Parkinson’s Disease

**DOI:** 10.3390/ijms27219688

**Published:** 2026-10-29

**Authors:** Zumeng Wang, Zhuoting Li, Meiling Wang, Lin Li, Kah-Leong Lim, Wenli Cui

**Affiliations:** 1 School of Basic Medical Sciences, Shanxi Medical University, NO. 56 Xinjian South Rd, Taiyuan 030001, China; ifezmwang@163.com (Z.W.);; 2 Key Laboratory of Cellular Physiology, Ministry of Education, Shanxi Medical University, Taiyuan 030001, China; 3 State Key Laboratory of Flexible Electronics (LoFE) & Institute of Flexible Electronics (IFE), Xiamen University, Xiamen 361000, Chinaifelli@xmu.edu.cn (L.L.); 4 Lee Kong Chian School of Medicine, Nanyang Technological University, 11 Mandalay Road, Singapore 308232, Singapore

**Keywords:** Parkinson’s disease, perivascular macrophages, single-nucleus RNA sequencing, Mendelian randomization, neurovascular remodeling

## Abstract

Parkinson’s disease (PD) is a common neurodegenerative disorder characterized by progressive neurovascular dysfunction and neuroinflammation, in which perivascular macrophages (PVMs) serve as key immune sentinels that regulate cerebrovascular homeostasis and disease progression. However, the transcriptional remodeling, genetic susceptibility, and functional mechanisms of PVMs in the pathogenesis of PD remain unclear. In this study, we integrated human midbrain single-nucleus RNA sequencing, genetic association datasets, and transcriptomic analyses to characterize PVM alterations in PD. Through multi-omics analyses, including Mendelian randomization, colocalization, and trajectory inference, we identified PD-associated PVM genes and regulatory pathways, which were further validated in a rotenone-induced PD model. We found extensive transcriptional remodeling of PVMs in PD, manifested by inflammatory activation, altered complement signaling, and aberrant neurovascular interactions. Genetic analyses identified *HLA-DRA*, *OSBPL3*, and *RIN3* as PD-associated PVM genes that were enriched in PVMs and exhibited dynamic changes during disease progression. Functional analyses suggested that these genes are involved in immune regulation, lipid metabolism, and intracellular trafficking. The rotenone-induced model further confirmed the upregulation of *Osbpl3* and *Rin3*. These findings reveal that PVM-centered neurovascular immune remodeling is a feature of PD and identify *HLA-DRA*, *OSBPL3*, and *RIN3* as candidate genes supported by genetic evidence, linking PVM dysfunction to PD susceptibility.

## 1. Introduction

Parkinson’s disease (PD) is the most common neurodegenerative movement disorder, characterized by progressive loss of dopaminergic neurons in the substantia nigra pars compacta and presence of abnormal α-synuclein aggregation [1,2]. There are more than 8.5 million PD patients worldwide, which is expected to increase with the global aging population [3]. The molecular mechanism underlying PD pathogenesis remains to be elucidated. Increasing evidence shows that neuroinflammation, immune dysregulation, and neurovascular dysfunction play pivotal roles in PD occurrence and progression [4].

Regarding neuroimmunity, previous studies mainly focused on microglia and astrocytes, which participate in inflammatory cytokine secretion and α-synuclein clearance [5,6]. More recently, perivascular macrophages (PVMs) have earned more attention due to their crucial role in regulating immune surveillance, phagocytic clearance, and antigen presentation [7,8,9]. Under physiological conditions, PVMs maintain homeostasis at the brain borders [10]; however, the detection of disease-associated α-synuclein immunoreactivity in PVMs from postmortem PD and dementia with Lewy bodies brains suggests a direct involvement in pathology [11]. In the presence of pathological α-synuclein, PVMs and other border-associated macrophages expand and adopt a disease-associated state characterized by enhanced antigen presentation and chemokine signaling. These activated cells promote CD4^+^ T-cell recruitment and MHC II-dependent neuroinflammation, thereby contributing to dopaminergic neurodegeneration [12]. These findings highlighted the role of PVMs in PD. However, the transcriptional states, intercellular communication features, and correlation with featured PD susceptibility genes remain poorly defined.

Single-cell and single-nucleus transcriptomics enabled definition of disease-associated cell type proportions and cell-specific gene expression signatures. However, differential expression genes (DEGs) alone could not determine their causal pathogenic effect on diseases [13]. Mendelian randomization (MR) used genetic variants as instrumental variables to assess potential causal associations between gene expression and disease risk [14]. Furthermore, colocalization and reverse MR could assess shared genetic signatures and causal association directions [15]. Integrating these approaches can pinpoint genetically validated candidate genes and unravel their cell-specific expression profiles.

In this study, to reveal the role and mechanism of PVMS in PD pathogenesis, we carried out integrating analysis of PD patients’ midbrain single-nucleus RNA (snRNA) sequencing, blood-derived cis-eQTL data, PD genome-wide association study (GWAS) summary statistics, and bulk substantia nigra transcriptomic data. We firstly characterized PD-associated PVM-specific gene alterations and intercellular communication, and defined their functionality, temporal dynamics along pseudotime, and immune infiltration. Then the screened PD susceptibility genes of PVMs were further validated in the in vitro and in vivo PD models. The integrative analysis prioritized *HLA-DRA*, *OSBPL3*, and *RIN3* as PD-associated PVM-enriched genes. Functional analyses demonstrated that those genes were involved in immune regulation, lipid metabolism, and intracellular trafficking, which are well-accepted PD risk factors. This study not only throws light on the molecular mechanisms of PD pathogenesis but also provides one feasible strategy to screen out disease-associated genes by integrating multiple omics analysis.

## 2. Results

### 2.1. Transcriptomic Remodeling of Midbrain Cells in PD

**Figure 1:**
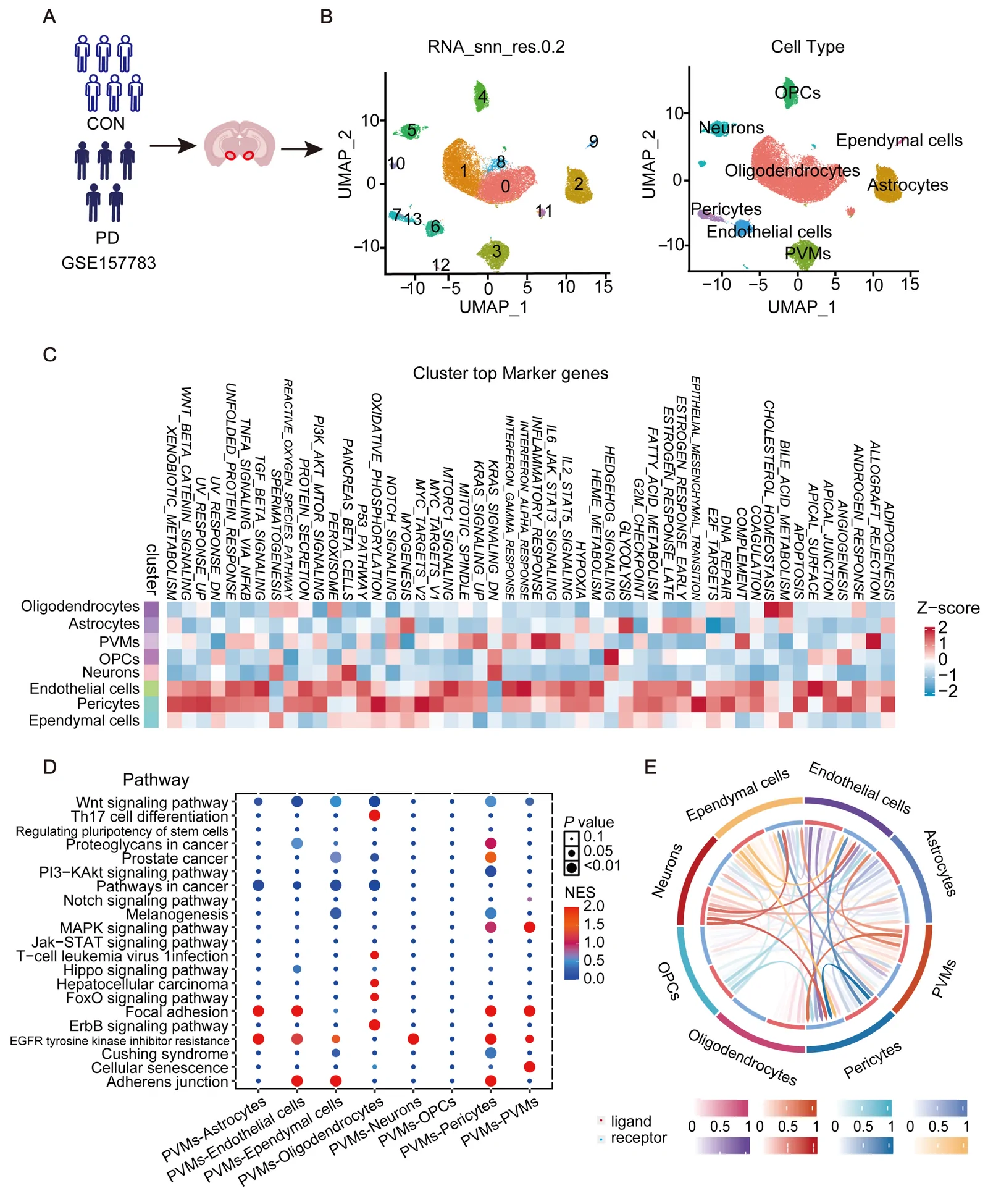
snRNA-seq-based dissection of cell subpopulations, characteristic pathways, and intercellular communication in PD midbrain. (**A**) Schematic of experimental design for dataset GSE157783 analyzed in this study, which includes midbrain tissue samples from PD and CON. PD: Parkinson’s disease; CON: neurologically normal controls. Red circles indicate the substantia nigra, the analyzed brain region. In panel B, numbers represent cluster IDs. (**B**) UMAP dimensionality reduction visualization of snRNA-seq data; left: original clustering; right: cell type annotation. (**C**) Heatmap of signaling pathway activities associated with the top marker genes in each cell type, with colors representing Z-scores. (**D**) Bubble plot of GSEA enrichment for PVMs versus all other cell types; dot size reflects the *p* value, and color indicates the normalized enrichment score (NES). (**E**) Chord diagram of ligand–receptor interactions among midbrain cell types, illustrating the strength of intercellular communication between different cell types.

To characterize cellular composition and transcriptomic signatures of PD midbrain, a publicly available snRNA-seq dataset comprising midbrain samples from five PD patients and six neurologically normal controls was analyzed. After Harmony integration and unsupervised clustering, eight midbrain cell subpopulations were identified and annotated: astrocytes, endothelial cells, ependymal cells, neurons, oligodendrocyte precursor cells (OPCs), oligodendrocytes, pericytes, and PVMs (Figure 1A, Supplementary Figure S1A–F). The PVM subpopulation was annotated based on the co-expression of macrophage lineage marker genes (*CSF1R* and *C1QB*) and the PVM-specific marker *CD163* (Supplementary Figure S1G). Cell-type-specific differential expression analysis revealed heterogeneous transcriptional responses among the major midbrain subpopulations under PD conditions (Figure 1B, Supplementary Figure S1H). At the donor level, no gene passed FDR < 0.05, consistent with the limited donor number and low nuclei counts in some donors (Table S1); among the prioritized genes, *OSBPL3* showed nominal upregulation (*p* = 0.015), in agreement with the cell-level results. Vascular-associated cells, including endothelial cells, pericytes, and PVMs, underwent pronounced disease-related transcriptional reprogramming. Metabolic pathway activity by cell subtype is shown in Figure 1C, with PVMs showing higher activity in Hallmark allograft rejection, IL-6–JAK–STAT3 signaling, and inflammatory response pathways (Figure 1C).

To further investigate the regulatory role of dysregulated intercellular communication in midbrain microenvironment remodeling in PD, a ligand–receptor interaction network among the cell subpopulations was constructed, and the potential intercellular signaling patterns within the midbrain were characterized. CellCall analysis of intercellular communication revealed that PVMs interacted with each cell subtype through multiple ligand–receptor pathways in a bidirectional manner (Figure 1D,E). Genes involved in PVM–endothelial cell and PVM–pericyte communication were enriched in cell adhesion processes, including focal adhesion and adherens junction, whereas those associated with PVM–oligodendrocyte communication were enriched in Th17 cell differentiation (Figure 1D). Together, these findings suggested that PVMs engage in extensive bidirectional communication within the midbrain microenvironment and contribute to inflammatory and metabolic reprogramming in PD.

### 2.2. Transcriptome-Wide Mendelian Randomization Identified Candidate Genes Associated with PD Risk

**Figure 2:**
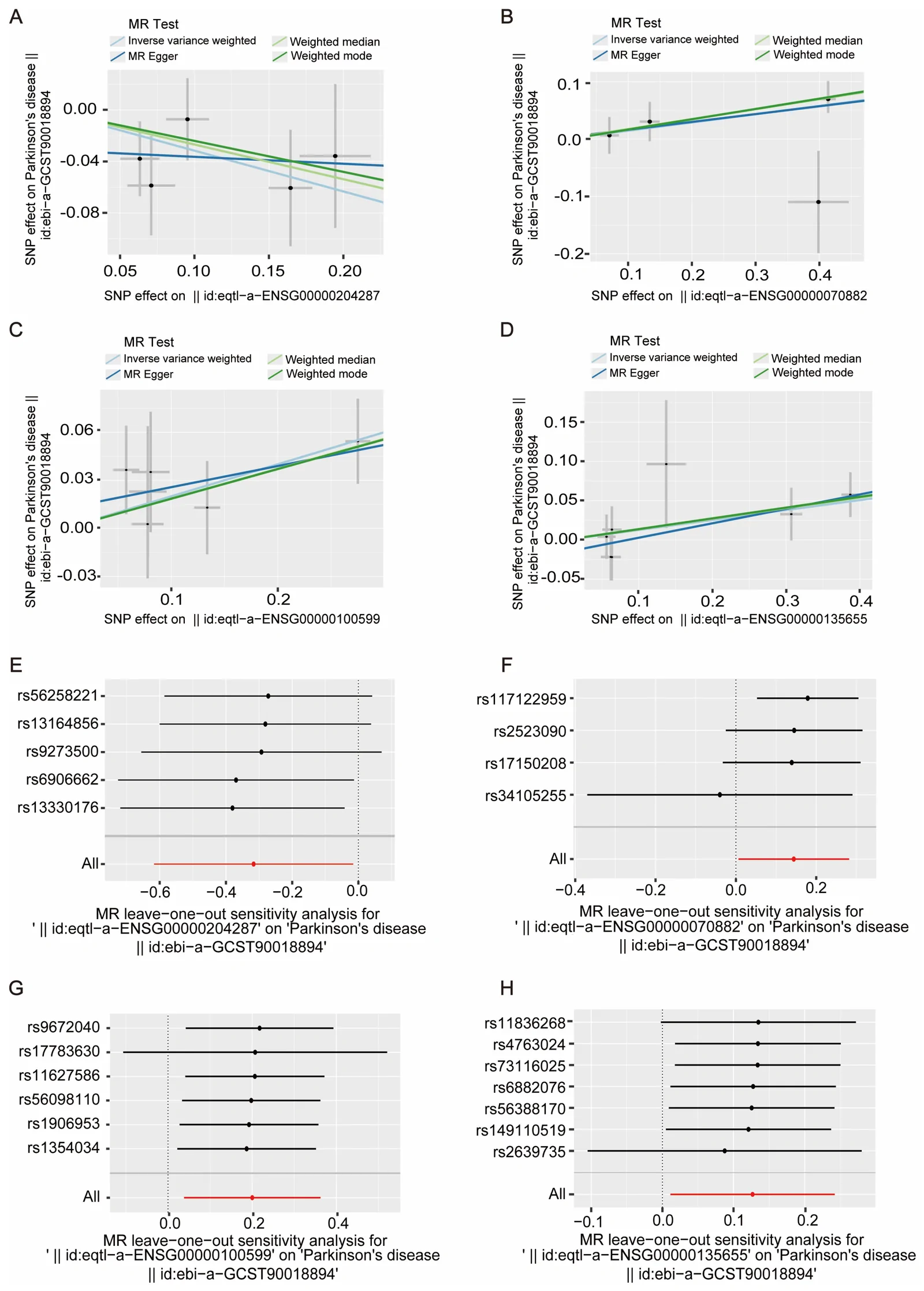
Two-sample MR evaluating associations between PD risk and genetically predicted expression of candidate genes. (**A**–**D**) MR scatter plots showing SNP effect sizes on gene expression exposure against SNP effect sizes on PD risk outcome for *HLA-DRA*, *OSBPL3*, *RIN3*, and *USP15*. Fitted lines for IVW, weighted median, weighted mode, and MR–Egger models are displayed. (**E**–**H**) Leave-one-out sensitivity analysis forest plots for the four genes. Each row represents an MR effect estimate omitting one instrumental-variable SNP; the “All” row denotes the overall incorporation of all IVs, testing for bias driven by a single outlier instrumental variable.

To identify potential causal genes for PD, a transcriptome-level two-sample MR analysis was performed. Summary-level cis-eQTL data from eQTLGen blood were used as exposure, and PD GWAS summary statistics comprising 2638 cases and 477,380 controls served as outcome. The IVW method was employed as the primary analysis to evaluate associations between genetically predicted gene expression and PD risk, supplemented by a series of sensitivity analyses. Eighteen genes with valid cis-eQTL instruments were tested and are reported as exploratory candidates (Table S2); their *p* values are unadjusted for multiple testing. Genetically predicted higher expression of *ARHGAP22*, *CTSB*, *HLA-DRA*, *LPAR6*, *PELI1* and *TBC1D1* was associated with lower PD risk, whereas higher expression of *CYTH4*, *GPNMB*, *ITGAX*, *LCP1*, *KYNU*, *LRRK2*, *OSBPL3*, *PREX1*, *RCSD1*, *RIN3*, *SAT1*, and *USP15* was associated with higher PD risk (Figure 2A, Supplementary Figure S2A). Leave-one-out analysis indicated that for genes with multiple instrumental variables, the association signals were not driven by any single instrumental variable. Cochran’s Q test and the MR–Egger intercept test revealed no significant heterogeneity or directional horizontal pleiotropy, suggesting that MR estimates were minimally influenced by these biases (Figure 2B, Supplementary Figure S2B).

### 2.3. Colocalization and Reverse MR Prioritize HLA-DRA, OSBPL3 and RIN3 as Candidate PD-Associated Genes

**Figure 3:**
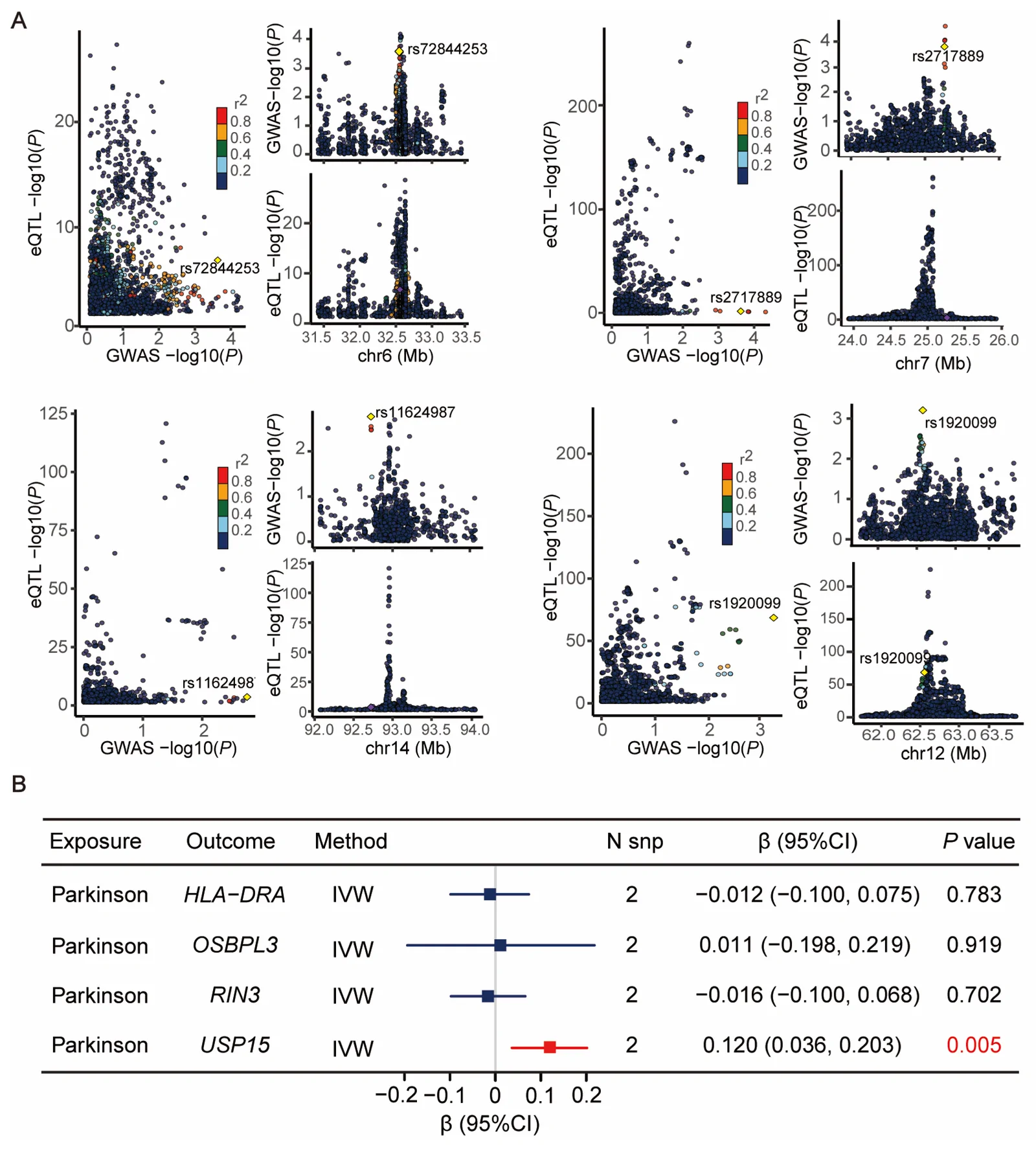
eQTL-reverse-GWAS colocalization analysis. (**A**) eQTL–GWAS colocalization for *HLA-DRA* (rs72844253), *OSBPL3* (rs2717889), *RIN3* (rs11624987), and *USP15* (rs1920099). Each panel includes scatter plots of GWAS versus cis-eQTL-log10(*p*) and locus-specific regional Manhattan plots highlighting the lead co-localized variant for each gene. (**B**) Reverse-MR forest plot showing IVW-derived β with 95% confidence intervals; β, effect on gene expression per unit increase in log-odds of PD liability.

To assess whether cis-eQTL and PD GWAS associations originate from the same conserved causal variant, an eQTL–GWAS colocalization analysis was performed. Four genes (*HLA-DRA*, *OSBPL3*, *RIN3*, and *USP15*) were identified for which there was strong evidence (PP.H4 > 0.90) that their cis-eQTLs and PD GWAS signals are driven by the same conserved causal variant (Figure 3A). Subsequently, a reverse MR analysis was conducted with PD genetic susceptibility as the exposure and genetically predicted expression levels of *HLA-DRA*, *OSBPL3*, *RIN3* and *USP15* as the outcome. No evidence of a reverse association was observed for *HLA-DRA*, *OSBPL3*, or *RIN3*. By contrast, a positive association was detected between PD genetic susceptibility and *USP15* expression (*p* = 0.005), suggesting that the *USP15* association may partly reflect an effect of PD liability on gene expression (Figure 3B). Accordingly, *HLA-DRA*, *OSBPL3* and *RIN3* were prioritized for subsequent functional and cellular analyses.

*HLA-DRA* (Major Histocompatibility Complex, class II, DR Alpha) is located at the MHC class II locus on 6p21.3 and encodes the α subunit of *HLA-DR* class II antigen molecule. The *HLA-DR* complex is expressed on the surface of antigen-presenting cells, and interacts with T-cell receptors to mediate antigen presentation, thereby regulating immune homeostasis in both the periphery and the central nervous system [16]. Specific *HLA-DRA* alleles are associated with differential PD susceptibility: HLA-DRA*01:01:01 is protective, whereas HLA-DRA*01:01:02 is associated with increased PD risk [17,18]. *HLA-DRA* also facilitates vesicle-mediated α-synuclein release, with PD risk-associated variants showing greater export capacity [19]. The observed upregulation of *HLA-DRA* in PD PVMs may therefore indicate enhanced antigen presentation and α-synuclein-associated immune responses.

*RIN3* (Ras and Rab interactor 3) encodes a Rab5-specific guanine nucleotide exchange factor involved in early endocytic trafficking and protein clearance [20,21]. In PD, *RIN3* hypomethylation in peripheral blood is associated with mild cognitive impairment, and its expression is enriched in disease-associated microglia [21,22]. Given that endolysosomal sorting and Rab GTPase signaling are established contributors to PD pathogenesis, its upregulation in PVMs may reflect altered Rab5-dependent endosomal trafficking and protein clearance, potentially contributing to innate immune dysfunction [23,24].

*OSBPL3* (oxysterol-binding protein-like 3) encodes a lipid-transfer protein involved in intracellular cholesterol transport and lipid homeostasis [25]. Although *OSBPL3* is reportedly downregulated in bulk PD brain tissue [26], we observed its upregulation in PD PVMs, indicating cell-type-specific regulation. This increase may reflect altered lipid metabolism or IRE1/RIDD signaling and contribute to PVM-mediated neuroinflammation [27,28,29]. Collectively, these findings implicated dysregulated antigen presentation, endosomal trafficking, and lipid homeostasis in PVM dysfunction in PD.

### 2.4. Functional Analyses Link Prioritized PD-Associated Genes to Immune, Metabolic, and Proteostatic Pathways

**Figure 4:**
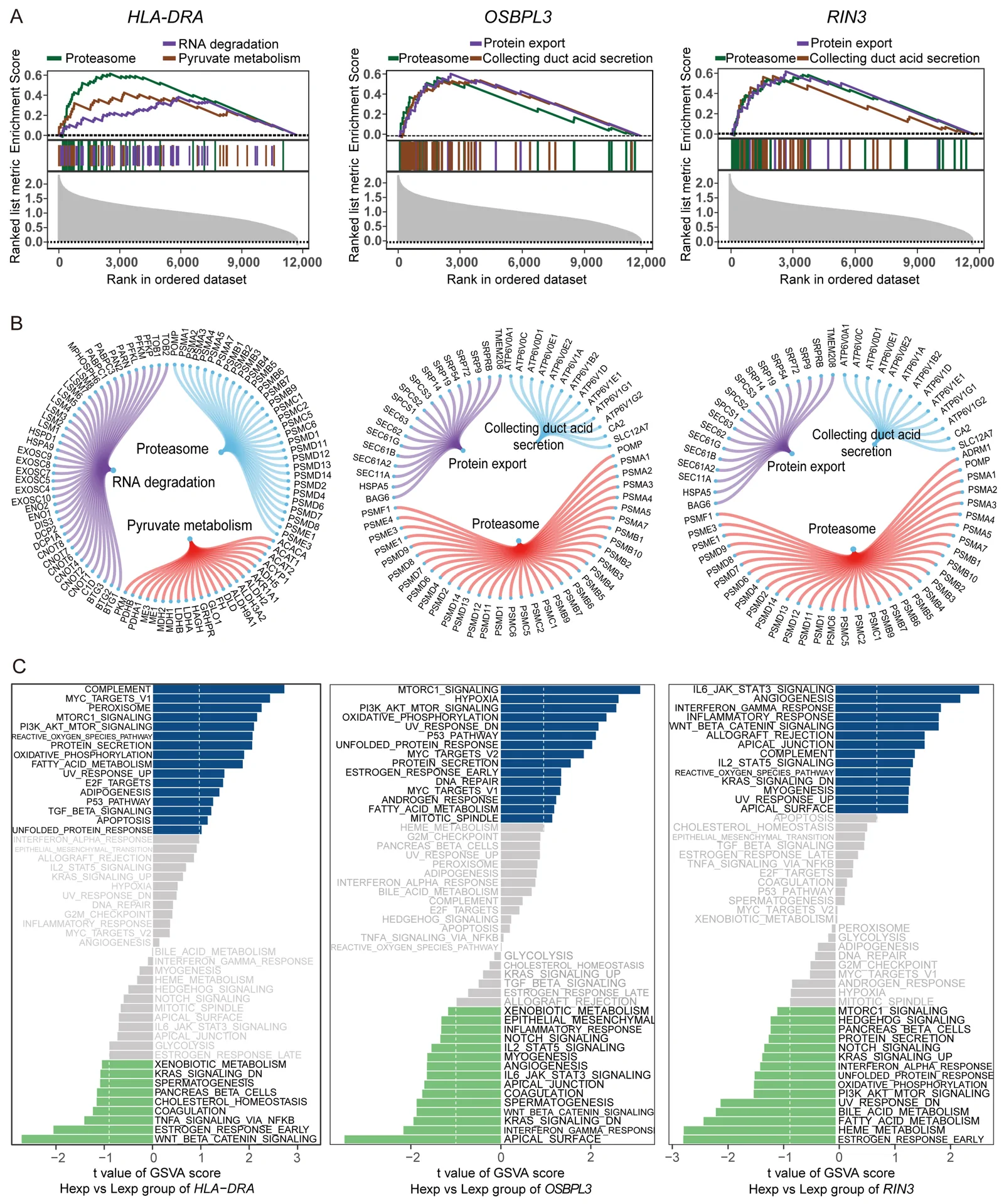
Pathway-level enrichment analyses for PD candidate genes using GSEA and GSVA. (**A**) GSEA running-score plots for *HLA-DRA*, *OSBPL3*, and *RIN3*, illustrating enrichment profiles of representative hallmark pathways. (**B**) Circus plots depicting the gene–pathway interaction relationships inferred from GSEA results of the three candidate genes. (**C**) GSVA comparison of pathway activity between high- and low-expression groups for each gene. Horizontal bars show t-statistics derived from GSVA scores. Blue, green, and grey bars indicate significantly upregulated, significantly downregulated, and non-significant pathways, respectively.

To further investigate biological mechanisms associated with the prioritized genes, we performed GSEA and GSVA based on *HLA-DRA*, *OSBPL3*, and *RIN3* expression. GSEA showed that genes correlated with the three candidates were enriched in proteostasis- and metabolism-related pathways. Genes positively associated with *HLA-DRA* were enriched in the proteasome, pyruvate metabolism, and RNA degradation pathways (Figure 4A,B). Both *OSBPL3* and *RIN3* exhibited shared enrichment for proteasome-related pathways, together with protein export and collecting duct acid secretion pathways (Figure 4A,B), suggesting potential roles in intracellular protein processing and proteostasis regulation. GSVA further demonstrated gene-specific pathway alterations: *HLA-DRA* was linked to immune activation and complement signaling, *OSBPL3* correlated with mTOR-related metabolic regulation and mitochondrial pathways and *RIN3* associated with inflammatory signaling and oxidative stress responses (Figure 4C). Together, these findings suggest that *HLA-DRA*, *OSBPL3*, and *RIN3* may contribute to PD pathogenesis through regulation of protein homeostasis, neuroimmune activation, and metabolic dysfunction.

### 2.5. MR-Prioritized PD-Associated Genes Are Associated with the Immune Context of the PD Substantia Nigra

**Figure 5:**
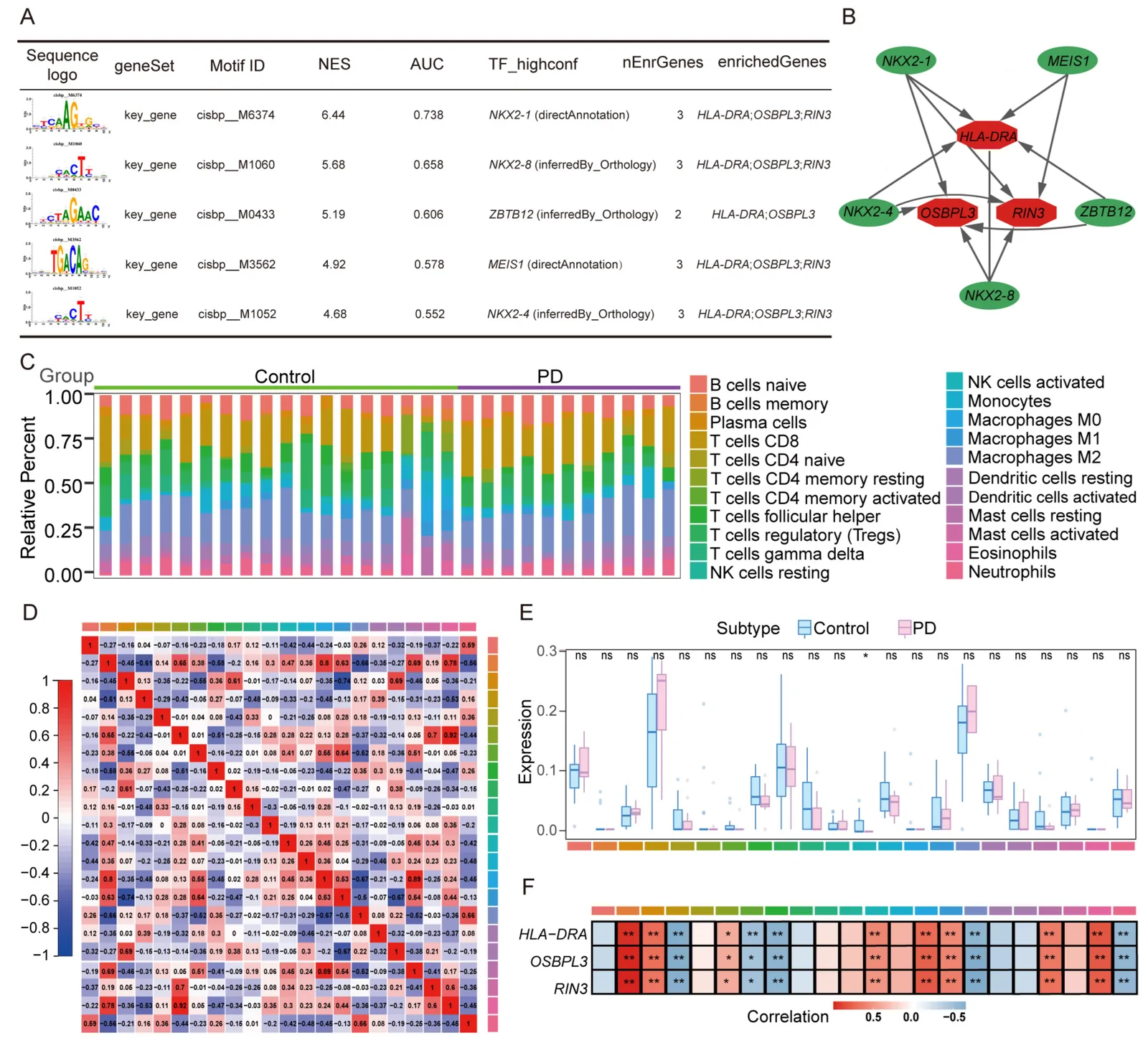
Transcription-factor regulatory profiling of candidate genes and immune cell infiltration analysis in the PD microenvironment. (**A**) Cis-target motif enrichment for *HLA-DRA*, *OSBPL3*, and *RIN3*, listing motif metrics and target genes. (**B**) Predicted regulatory network between candidate genes and upstream transcription factors. Red polygons indicate target genes; green ellipses indicate transcription factors. (**C**) Stacked bar graph showing relative proportions of 22 immune cell subtypes in control and disease groups. (**D**) Heatmap of pairwise correlations among 22 immune cell subsets. (**E**) Heatmap of pairwise correlations among 22 immune cell subsets. (**F**) Heatmap displaying correlation coefficients between candidate-gene expression and immune cell abundance. (**D**–**F**) use the same legend as (**C**) for explanation. ns, not significant; * *p* < 0.05; ** *p* < 0.01.

To further characterize the regulatory context of *HLA-DRA*, *OSBPL3*, and *RIN3*, we performed motif-based transcription factor enrichment analysis. Multiple enriched motifs and their corresponding transcription factors were identified. The top five annotated TFs span three classes: *NKX2-1*, *NKX2-8*, and *NKX2-4* (NKX2 homeobox), *MEIS1* (TALE-homeobox), and *ZBTB12* (BTB/POZ zinc-finger). NKX2-family factors each target all three candidate genes, suggesting potential shared upstream transcriptional regulation (Figure 5A,B). We next investigated their immune context through immune cell deconvolution and correlation analyses. PD patients exhibited distinct immune landscape alterations, with a significant reduction in activated NK cells, whereas other immune populations showed no marked changes (Figure 5C,E). Correlation analysis revealed extensive immune network remodeling involving macrophage, T-cell, B-cell, and granulocyte populations (Figure 5D). Furthermore, *HLA-DRA*, *OSBPL3* and *RIN3* expression was broadly associated with diverse immune cell states, including lymphoid and myeloid populations (Figure 5F), suggesting that these genes are embedded within a complex immune regulatory network. Collectively, these findings link PD genetic susceptibility with immune landscape remodeling and provide additional evidence for immune dysregulation in PD pathogenesis.

### 2.6. MR-Prioritized PD Susceptibility Genes Define Disease-Associated PVM States

**Figure 6:**
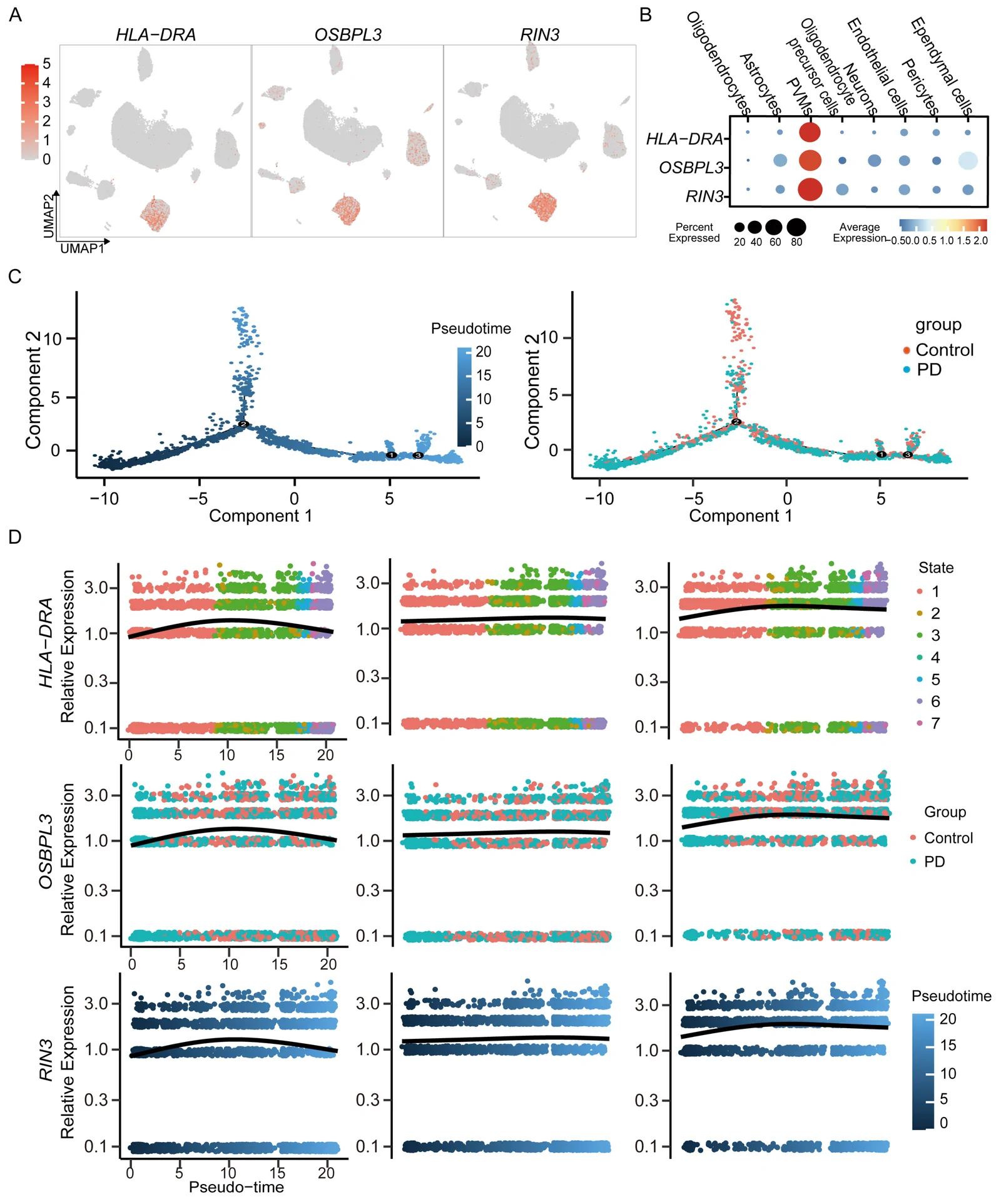
Single-cell expression and pseudotime trajectory analysis of the three prioritized PD candidate genes. (**A**) UMAP feature plot showing single-cell expression patterns of *HLA-DRA*, *OSBPL3*, and *RIN3* across midbrain cell populations. (**B**) Dot plot displaying average expression and the percentage of cells expressing each candidate gene across annotated midbrain cell types. Dot size corresponds to the fraction of expressing cells; color intensity denotes mean gene expression. (**C**) Pseudotime trajectories reconstructed from human midbrain snRNA-seq data. Cells are colored by pseudotime (**left**) or disease status (control versus PD, **right**). (**D**) Dynamic gene expression profiles of *HLA-DRA*, *OSBPL3*, and *RIN3* along the inferred pseudotime axis for control and disease groups. Black lines represent smoothed trend curves of gene expression.

To determine the cellular localization and dynamic regulation of MR-prioritized PD susceptibility genes, we integrated single-cell expression profiling with pseudotime trajectory analysis of *HLA-DRA*, *OSBPL3* and *RIN3*. UMAP visualization and dot plot analyses revealed that these genes were preferentially expressed in PVMs compared with other brain cell types (Figure 6A,B). To further explore PVM state transitions in PD, we used Monocle to order PVM cells according to pseudotime based on their transcriptional profiles. Using the earliest PVM state as the root, cells were ordered along a transcriptional continuum (Figure 6C). PD and control PVMs showed differential occupancy along the inferred transcriptional trajectory (Figure 6C), suggesting altered PVM state progression in PD. Differential expression analysis across pseudotime states identified transition-associated gene programs (Supplementary Figure S3). Notably, *HLA-DRA*, *OSBPL3* and *RIN3* showed dynamic expression patterns along the trajectory, linking genetically prioritized PD susceptibility genes to disease-associated PVM state remodeling (Figure 6D). Collectively, single-cell profiling and pseudotime analysis demonstrate that *HLA-DRA*, *OSBPL3* and *RIN3* are preferentially localized to PVMs and associated with PD-related PVM transcriptional state remodeling, suggesting their potential involvement in PD-associated neuroimmune alterations.

### 2.7. Validation of HLA-DRA, OSBPL3, and RIN3 Alterations in Rotenone-Induced In Vivo and In Vitro PD Models

**Figure 7:**
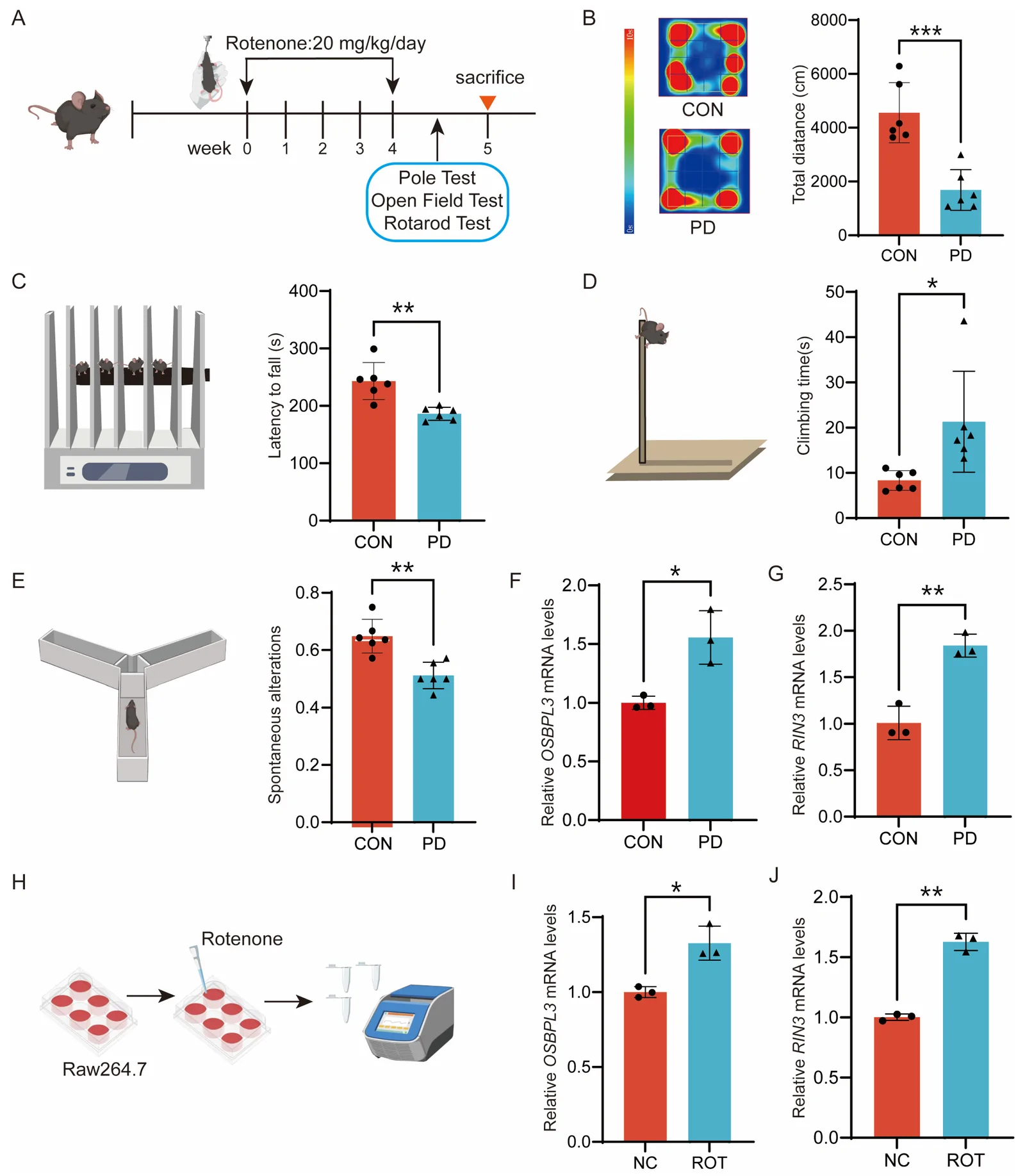
Molecular validation of key genes in PD mouse model and cell lines. (**A**) Schematic diagram of mouse PD model construction. (**B**) Total distance traveled by mice in the open-field test. (**C**) Latency to fall of the rotating rod test. (**D**) Climbing times of the climbing rod test. (**E**) Spontaneous alternation rate of the Y-maze test. (**F**,**G**) Relative mRNA levels of *Osbpl3* and *Rin3* in the substantia nigra of CON and PD mice. (**H**) Workflow for establishing the in vitro rotenone-treated neuronal PD model. (**I**,**J**) Relative mRNA levels of *Osbpl3* and *Rin3* in ROT-treated PVM-like and NC cells; CON: control mice; NC: negative control. * *p* < 0.05, ** *p* < 0.01, *** *p* < 0.001.

To validate expression of prioritized genes in experimental PD models, we established rotenone-induced in vivo and in vitro PD-like models. Rotenone-treated mice exhibited reduced locomotor activity in the open-field test (Figure 7A,B), decreased latency to fall in the rotarod test (Figure 7C), prolonged pole descent time (Figure 7D), and impaired spontaneous alternation performance in the Y-maze (Figure 7E), confirming the induction of PD-like behavioral deficits. qRT-PCR analysis of substantia nigra tissues showed that *Osbpl3* and *Rin3* were significantly upregulated, whereas *H2-Ea* (mouse orthologous gene of the human *HLA-DRA* gene) expression was undetectable (Figure 7F,G). We further examined these genes in cultured PVM-like cells exposed to rotenone. Consistently, rotenone treatment increased *Osbpl3* and *Rin3* expression (Figure 7H–J). Together, these findings indicate that *RIN3* and *OSBPL3* exhibited disease-associated dysregulation consistent with the direction of alteration observed in single-cell analyses.

## 3. Discussion

PD is characterized by concurrent neurovascular impairment and neuroimmune dysregulation, in which PVMs serve as a critical bridge linking cerebrovascular homeostasis and immune responses [30]. Recent single-cell and single-nucleus transcriptomic studies have revealed cell-type-specific alterations in dopaminergic neurons, microglia, astrocytes, and vascular cells, highlighting the multicellular nature of PD pathology [31,32,33]. However, the disease-specific transcriptional remodeling of PVMs and their genetic contribution to PD pathogenesis remain insufficiently elucidated. Here, by integrating human midbrain snRNA, bulk substantia nigra transcriptomics, MR, colocalization analysis, and experimental validation, we identified PVMs as a potentially important neurovascular immune population in PD and prioritized *HLA-DRA*, *OSBPL3*, and *RIN3* as genetically supported candidate genes associated with PD-related PVM states. These findings provide convergent evidence linking PD genetic susceptibility to PVM-associated neurovascular immune remodeling.

PVM-upregulated genes were enriched in inflammatory response, IL-6–JAK–STAT3 signaling, complement activation, and antigen-presentation-related pathways, consistent with the reported involvement of BAMs in α-synuclein-associated immune responses [34]. Inferred communication between PVMs and endothelial cells or pericytes, together with enrichment of focal adhesion and adherens junction pathways, further suggests that PVMs may participate in neurovascular remodeling in the PD midbrain [35,36]. Nevertheless, these interactions require spatial and functional validation.

MR analysis showed that higher *HLA-DRA* expression was associated with a lower risk of PD, whereas higher *OSBPL3* and *RIN3* expression was associated with increased risk. Colocalization analysis further supported the possibility that gene expression and PD GWAS signals share a common genetic basis, while reverse MR provided no clear evidence that PD genetic liability influenced the expression of these three genes. These findings support the prioritization of the three genes but do not eliminate potential pleiotropy or residual genetic confounding. Importantly, the instrumental variables were derived from blood cis-eQTL data, whose regulatory effects may not fully reflect those operating in the midbrain or PVMs. Therefore, these results should not be interpreted as evidence of PVM-specific causality and require validation using brain- or myeloid-cell-specific regulatory datasets.

The three candidate genes may represent complementary aspects of PD-associated PVM dysfunction. *HLA-DRA* mediates MHC class II-mediated antigen presentation and local adaptive immunity [37], though its inverse association with PD risk implies context-dependent effects. *OSBPL3* regulates lipid transport and membrane organization and may contribute to the metabolic adaptation of PVMs [38], and *RIN3* controls Rab-dependent endosomal-trafficking phagolysosomal homeostasis [39]. Functional enrichment and pseudotime analyses linked these genes to immune, metabolic, and proteostatic programs; immune deconvolution revealed a selective reduction in activated NK cell signatures rather than broad immune cell remodeling. Consistent with a shared regulatory basis, the predicted upstream regulators span three TF classes. *NKX2-1*, *NKX2-8*, and *NKX2-4* (*NKX2* homeobox) each target all three genes, identifying *NKX2* factors as the dominant shared regulators; a PD-associated SNCA variant (rs7684318) is predicted to reduce *NKX2*-family bindingin the midbrain, putamen, and caudate, implicating *NKX2*-driven SNCA regulation in dopaminergic dysfunction [40]. *MEIS1* (TALE-homeobox) also targets all three genes and, as a risk gene regulating dopaminergic and iron-metabolic functions in substantia nigra neurons, supports shared neurodegenerative mechanisms with PD [41]. *ZBTB12* (BTB/POZ zinc-finger) targets only *HLA-DRA* and *OSBPL3*, suggesting a gene-specific regulatory layer. However, these findings remain correlational. Pseudotime does not represent actual temporal progression, and the LM22 matrix cannot distinguish PVMs from microglia or other brain-resident myeloid cells. The predicted regulators are likewise sequence-based inferences requiring validation. Thus, these analyses support coordinated immuno-metabolic remodeling of PVMs in PD but do not establish pathway activation, PVM-specific abundance changes, or causal regulatory relationships.

*Osbpl3* and *Rin3* were consistently upregulated in rotenone-induced in vivo and in vitro models, providing cross-system support for their PD-associated expression changes. However, because rotenone induces mitochondrial complex I inhibition, oxidative stress, and cellular injury [42,43], these alterations may reflect pathogenic susceptibility, downstream stress responses, or compensatory adaptation. Moreover, the experimental measurements were not PVM-specific. PVM-targeted perturbation and rescue experiments, combined with PVM–endothelial cell or PVM–dopaminergic neuron coculture, will therefore be required to determine their effects on barrier integrity, inflammation, phagocytosis, and neuronal survival. Because the selected murine MHC class II readout was below the reliable detection limit, the *HLA-DRA*-associated antigen-presentation program should be evaluated using additional murine MHC class II markers or human PVM-like cellular systems.

This study integrated human midbrain single-nucleus transcriptomics, genetic causal inference, bulk transcriptomic validation, and experimental PD models to characterize PVM-associated molecular alterations and prioritize candidate genes. However, several limitations should be acknowledged. First, the single-nucleus transcriptomic dataset contained a limited number of postmortem samples, and the markers used to annotate PVMs, including *CSF1R, C1QB*, and *CD163*, may overlap with other BAM and myeloid-cell states. Second, the MR analyses relied on blood-derived cis-eQTLs and therefore could not establish brain- or PVM-specific genetic effects. Third, cell–cell communication, pathway enrichment, motif analysis, immune deconvolution, and pseudotime reconstruction were computational inferences that require spatial and functional validation. Fourth, bulk transcriptomic data could not distinguish altered cellular composition from cell-intrinsic transcriptional changes. Finally, although *Osbpl3* and *Rin3* expression changes were reproduced in experimental PD models, these analyses were not PVM-specific, did not include direct genetic perturbation or rescue experiments, and did not confirm protein-level change.

## 4. Materials and Methods

### 4.1. Single-Nucleus RNA Sequencing and Bioinformatic Analysis

#### 4.1.1. Data Collection and Preprocessing

Human midbrain snRNA-seq data were obtained from the GEO database (https://www.ncbi.nlm.nih.gov/geo/ (accessed on 20 September 2024)), including dataset GSE157783 with 11 samples (5 PD and 6 normal controls), which was generated from postmortem tissue and is well suited to preserving PVMs [44,45]. Bulk substantia nigra transcriptomic data from GSE20292 (GPL96 platform; 11 PD and 18 controls) were used for immune infiltration analysis.

The snRNA-seq expression matrix was processed using the R (v4.3.0) and Seurat package (v4.3.0). Cells were filtered based on quality metrics including nFeature_RNA, nCount_RNA, mitochondrial transcript proportion (percent.mt), and ribosomal transcript proportion (percent.ribo) [46]. Cells with nFeature_RNA < 1000 or >5500, nCount_RNA < 1000 or >60,000, and percent.mt > 5% were excluded, and additional outliers were removed using a 3-median absolute deviation (MAD) criterion [47]. Doublets were identified and removed using DoubletFinder (v2.0.4).

The filtered datasets were normalized using NormalizeData, followed by identification of 2000 highly variable genes (HVGs) using FindVariableFeatures and scaling with regression of mitochondrial, ribosomal, and cell-cycle effects. PCA was performed, and the first 20 principal components were used for downstream analysis. Batch effects were corrected using Harmony (v1.1.0) with sample identity as the integration variable. UMAP visualization and graph-based clustering were performed using FindNeighbors and FindClusters with a resolution of 0.2. Cell types were annotated based on canonical marker genes, the CellMarker and PanglaoDB databases, published literature, and SingleR (v2.4.0) prediction [48].

For differential expression analysis, cell-level differential expression was performed using Seurat FindMarkers (Wilcoxon rank-sum test) and is reported as exploratory, as nuclei from the same donor are not independent observations. To account for this nested structure, a donor-level pseudobulk analysis was additionally performed using limma-voom (v3.58.1), in which PVM counts were aggregated within each donor and donors were treated as the biological replicates (5 PD vs. 6 controls), thereby collapsing nuclei into one observation per donor. Multiple testing was corrected using the Benjamini—Hochberg procedure (FDR < 0.05). The number of PVM nuclei retained per donor after quality control is summarized in Table S1; no additional minimum-nuclei threshold was imposed, as donor-level aggregation renders the analysis robust to uneven nuclei numbers. Marker genes for each cell subpopulation were identified using the FindAllMarkers function with thresholds of avg_log2FC > 1 and *p*_val_adj < 0.05, and the top five markers with the highest fold change were visualized using MarkerVolcano.

#### 4.1.2. Cell–Cell Communication and Functional Enrichment Analysis

Cell–cell communication analysis was performed using CellChat (v1.6.1) to infer ligand–receptor–transcription factor (L-R-TF) signaling networks based on gene expression profiles. Pathway enrichment analysis was conducted using GSEA and GSVA (v1.50.0). GSEA was performed using MSigDB gene sets (v7.0) with genes ranked according to differential expression statistics, and pathways with adjusted *p* < 0.05 were considered significant. GSVA was applied to quantify sample-level pathway activity based on predefined gene sets [49,50,51,52].

#### 4.1.3. Transcriptional Regulation and Immune Infiltration Analysis

Transcription factor activity was inferred using RcisTarget (v1.19.2) based on motif enrichment analysis. Motif-associated gene set enrichment was evaluated using the area under the recovery curve (AUC), and motifs with NES > 3 were considered significantly enriched. Immune cell composition was estimated using CIBERSORT (original web version, http://cibersort.stanford.edu/) based on the LM22 signature matrix containing 547 immune marker genes. The relative abundance of 22 immune cell subsets was inferred from bulk substantia nigra transcriptomic profiles, followed by correlation analysis between candidate genes and immune cell fractions [53].

#### 4.1.4. Pseudotime Trajectory Analysis

Cell-state transitions were investigated using Monocle3 (v2.30.0) pseudotime trajectory analysis. Cells were ordered along transcriptional trajectories based on gene expression similarity, and dynamic gene expression changes associated with cellular state transitions were identified [54,55].

### 4.2. Mendelian Randomization

#### 4.2.1. MR Study Design

A two-sample MR framework was used to evaluate the causal effects of genetically predicted gene expression on PD susceptibility. Blood-derived cis-eQTL summary statistics were used as exposure datasets, and PD GWAS summary statistics were used as outcome datasets.

#### 4.2.2. eQTL and PD GWAS Data Source

Blood-derived cis-eQTL summary statistics were obtained from the eQTLGen Consortium (https://www.eqtlgen.org), including SNP identifiers, genomic positions, effect alleles, effect sizes (β), standard errors, and effect allele frequencies. PD GWAS summary statistics were obtained from the IEU OpenGWAS database (ID: ebi-a-GCST90018894), including 2638 PD cases and 477,380 controls.

#### 4.2.3. Instrument Selection

For each candidate gene, SNPs associated with gene expression were selected as instrumental variables (IVs) using a locus-wide significance threshold (*p* < 1 × 10^−5^). Independent genetic instruments were obtained through LD clumping (r^2^ < 0.001, window size = 10,000 kb), and instrument strength was assessed using F-statistics (F > 10). Causal estimates were primarily derived using the inverse-variance-weighted (IVW) method, with MR–Egger regression, weighted median, and weighted mode analyses performed as sensitivity approaches. For genes with a single available IV, the Wald ratio method was applied [56]. The *p* < 1 × 10^−5^ threshold was applied solely for instrument selection and does not correct the gene-level tests. Eighteen genes with valid cis-eQTL instruments were tested by IVW Mendelian randomization, and gene-level *p* values are reported unadjusted (Table S2). Candidates were prioritized by sensitivity analyses (MR–Egger, weighted median, leave-one-out) and colocalization rather than by nominal significance. No association is claimed to reach genome-wide or FDR-level significance, and replication in independent cohorts is required.

#### 4.2.4. Sensitivity Analysis

The robustness of MR estimates was assessed using Cochran’s Q test for heterogeneity, MR–Egger intercept analysis for horizontal pleiotropy, and leave-one-out analysis to evaluate the influence of individual SNPs on causal estimates [57,58,59].

#### 4.2.5. Colocalization Analysis

Bayesian colocalization analysis was performed using the coloc (v5.2.3) framework by integrating cis-eQTL and PD GWAS summary statistics to determine whether gene expression and PD susceptibility shared causal variants. Posterior probabilities were calculated within a 100 kb genomic region surrounding the lead SNP across five hypotheses (PP.H0–PP.H4). PP.H3 indicates association of both traits with distinct causal variants, whereas PP.H4 represents a shared causal variant. A PP.H4 > 0.90 was considered strong evidence of colocalization [60,61]. Default prior probabilities of p1 = p2 = 1 × 10^−4^ and p12 = 1 × 10^−5^ were used (Table S3).

### 4.3. Statistical Analysis

All statistical analyses were performed using R software version 4.3.0. For MR analyses, causal inference was based on three core assumptions: relevance of genetic instruments to exposure, independence from potential confounders, and exclusion of alternative pathways between instruments and outcomes [62]. All statistical tests were carried out using a two-sided test, and *p* < 0.05 was considered statistically significant.

### 4.4. Animals

Male C57BL/6J mice (8–10 weeks old) were obtained from the Laboratory Animal Center of Shanxi Medical University and housed under standard conditions (22 ± 2 °C, 12 h light/dark cycle). All procedures were approved by the Institutional Animal Care and Use Committee of Shanxi Medical University (Approval No. SYXK-2024-0007) and performed according to NIH guidelines. Mice were randomly divided into two groups (*n* = 6/group). The rotenone group received daily oral gavage of rotenone (20 mg/kg) for 28 days, while the control group received an equal volume of vehicle (DMSO, Tween-80, and PEG-300 in PBS). Behavioral tests were performed during the fourth week. Mice were then anesthetized with isoflurane (2% induction, 1% maintenance in oxygen) and euthanized for brain tissue collection.

### 4.5. Behavioral Experiments

#### 4.5.1. Open-Field Test

The open-field test was performed to assess spontaneous locomotor activity. Mice were placed in the center of the arena and monitored for 5 min using a video-tracking system (SMART v3.0; Bioseb, Vitrolles, France). Total distance traveled was analyzed as an indicator of locomotor activity.

#### 4.5.2. Rotarod Test

Motor coordination was assessed using an accelerating rotarod (ZB-200; Chengdu Taimeng Software Co., Ltd., Chengdu, China). Mice were placed on the rotating rod, with the speed gradually increased from 4 to 40 rpm over 5 min, and latency to fall was recorded. Mice were trained for 3 consecutive days before testing, and the average latency from three trials was used for statistical analysis.

#### 4.5.3. Pole Test

Mice were placed on the top of a vertical wooden pole (50 cm in length and 1 cm in diameter) with a spherical top (2.5 cm in diameter) and allowed to descend freely(SA111; Jiangsu SANS Biological Technology Co., Ltd., Nanjing, China). The time required to reach the base of the pole was recorded. Each mouse underwent five trials, and the mean descent time was calculated for statistical analysis.

#### 4.5.4. Y-Maze

Y-maze test was performed to assess spatial working memory. Mice were placed in the center of a Y-shaped maze (XR-XY1032; Shanghai Xinruan Information Technology Co., Ltd., Shanghai, China) with three arms positioned at 120° and allowed to explore freely for 5 min. Arm entries and spontaneous alternations were recorded, and the alternation percentage was calculated.

### 4.6. Cell Culture

RAW264.7 macrophages (HySigen Biosciences, Suzhou, China, Cat#TCM-C766) were used as an in vitro macrophage model due to the lack of a well-characterized immortalized PVM cell line. RAW264.7 mouse macrophages were cultured in DMEM (Gibco, New York, NY, USA, Cat#C11995500BT) supplemented with 10% FBS (10099-141C) and 1% penicillin–streptomycin (Solarbio, Beijing, China, Cat#P1400) at 37 °C in a humidified 5% CO_2_ incubator (Thermo Fisher Scientific, Waltham, MA, USA). Cells were treated with rotenone (final concentration: 10 μM) for 18 h and then collected for qRT-PCR analysis.

### 4.7. Quantitative Real-Time PCR

Total RNA was extracted from brain tissues and cultured cells using RNAiso Plus (Takara, Shiga, Japan; Cat#9109) and reverse-transcribed into cDNA using the PrimeScript RT reagent kit (Takara, Shiga, Japan; Cat#RR047A). qRT-PCR was performed using the TB Green PCR reagent system (Takara, Shiga, Japan; Cat#RR820A), and relative gene expression was calculated using the 2^−ΔΔCt method with *Actb* as the internal control. Primer (synthesized by BGItech, Shenzhen, China) sequences are listed in Table S4.

## 5. Conclusions

This study identifies PVMs as a potentially important component of neurovascular immune remodeling in PD and prioritizes *HLA-DRA*, *OSBPL3*, and *RIN3* as candidate genes associated with distinct PVM-related processes, including antigen presentation, lipid metabolism, and endosomal trafficking. These findings provide an integrative cellular and molecular framework for investigating PVM dysfunction in PD, while PVM-specific functional studies are required to establish the causal roles and therapeutic relevance of these genes.

## Data Availability

The publicly available human midbrain snRNA-seq (GSE157783) and human substantia nigra bulk transcriptomic (GSE20292) data were obtained from the NCBI GEO database. As both are de-identified and publicly available, their secondary reanalysis was exempt from additional ethics approval under the regulations of the Ethics Committee of Shanxi Medical University. All datasets generated and analyzed during this study are included in this article and its Supplementary Materials. Further information is available from the corresponding authors upon reasonable request. The original code used for data processing and analysis is openly available on GitHub at https://github.com/bioinfo-Li/Single-cell-and-Mendelian-randomization (accessed on 17 October 2026).

## Supplementary Materials

The following supporting information can be downloaded at: https://www.mdpi.com/article/10.3390/ijms27219688/s1.
