# Supplementary material for: Single-Cell and Mendelian Randomization Analyses Reveal Novel Genetic Risk Genes of Perivascular Macrophages in Parkinson’s Disease
Source: Int J Mol Sci. 2026 Oct 29;27(21):9688. doi: 10.3390/ijms27219688 (PMC13763142; doi:10.3390/ijms27219688)
Supplement: Supplementary file 1 [file ijms-27-09688-s001.zip › ijms-4605949-supplementary.pdf]

International Journal of Molecular Sciences

Supplementary Materials for

Single-cell and Mendelian randomization analysis reveal novel genetic risk genes of perivascular macrophages in Parkinson's disease

Zumeng Wang[1,2,†], Zhuoting Li[1,2,†], Meiling Wang[3], Lin Li[3], Kah-Leong Lim[4*], Wenli Cui[1,2*]

†These authors contributed equally to this work

correspondence to: cuiwenli1@sxmu.edu.cn; kahleong.lim@ntu.edu.sg

This PDF file includes:

Figure S1. snRNA-seq-based dissection of cell subpopulations, characteristic pathways, and intercellular communication in PD midbrain.

Figure S2. Two-sample MR evaluating associations between PD risk and genetically predicted expression of candidate genes.

Figure S3. Single-cell expression and pseudotime trajectory analysis of the three prioritized PD candidate genes.

Table S1. Number of PVM nuclei retained per donor after quality control, used as the donor-level biological replicates in the pseudobulk differential expression analysis.

Table S2. Inverse-variance weighted (IVW) two-sample Mendelian randomization results for 18 cis-eQTL instruments prioritized from PVM differential expression. β

and 95% CI are given on the log-odds scale; OR = exp(β). All 18 associations are treated as exploratory candidates and further ranked by sensitivity analyses and colocalization.

Table S3. Colocalization statistics for the four prioritized loci, using default coloc priors (p1 = p2 = 1×10^{-4}, p12 = 1×10^{-5}). PP.H4 is the posterior probability that the eQTL and PD GWAS signals share the same causal variant at the lead SNP. PP.H3 and the full variant count within each region are to be completed against the R coloc summary output.

Table S4. The primer sequences used for mouse genes.

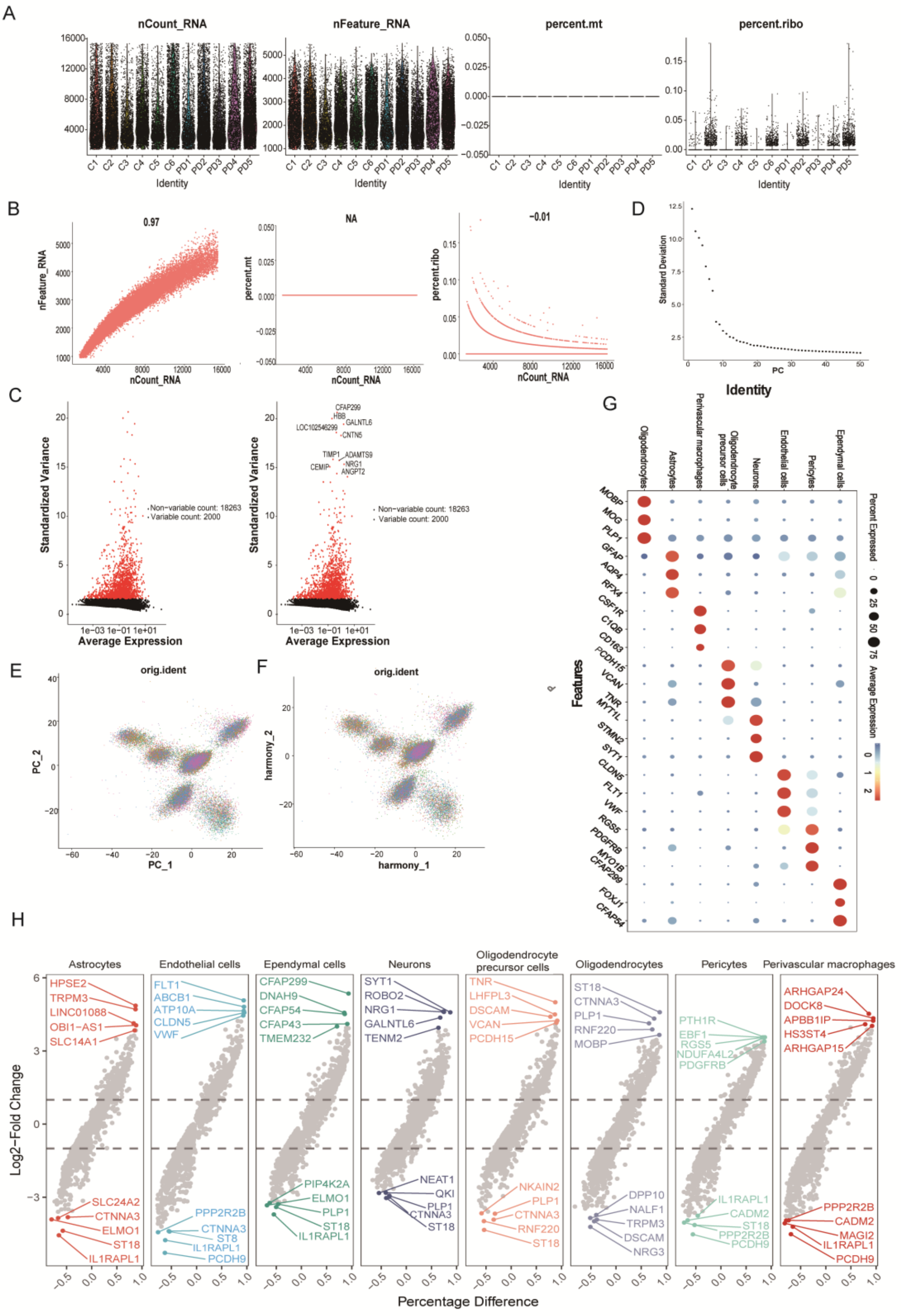

Supplementary Figure S1 snRNA-seq-based dissection of cell subpopulations and characteristic molecular features in PD midbrain. (A–F) Single-cell quality control and dimensionality reduction analyses. **A** Distributions of UMI counts, detected gene numbers, mitochondrial and ribosomal read percentages per cell. **B**

Pearson correlations among quality-control metrics. **C** Highly variable genes (HVGs) are identified via the FindVariableFeatures function. Red dots denote the selected HVGs. **D** Selection of statistically significant principal components based on PCA standard deviations. **E** PCA visualization without batch correction, coloured by sample. **F** Batch-effect removal using Harmony for cell embedding, coloured by sample. **G** Bubble plot showing marker gene expression across cell types. **H)** Volcano plots illustrating top differentially expressed marker genes for each cell subtype (FindAllMarkers, avg_log2FC > 1, p_val_adj < 0.05); top 5 fold-change markers are labelled.

A

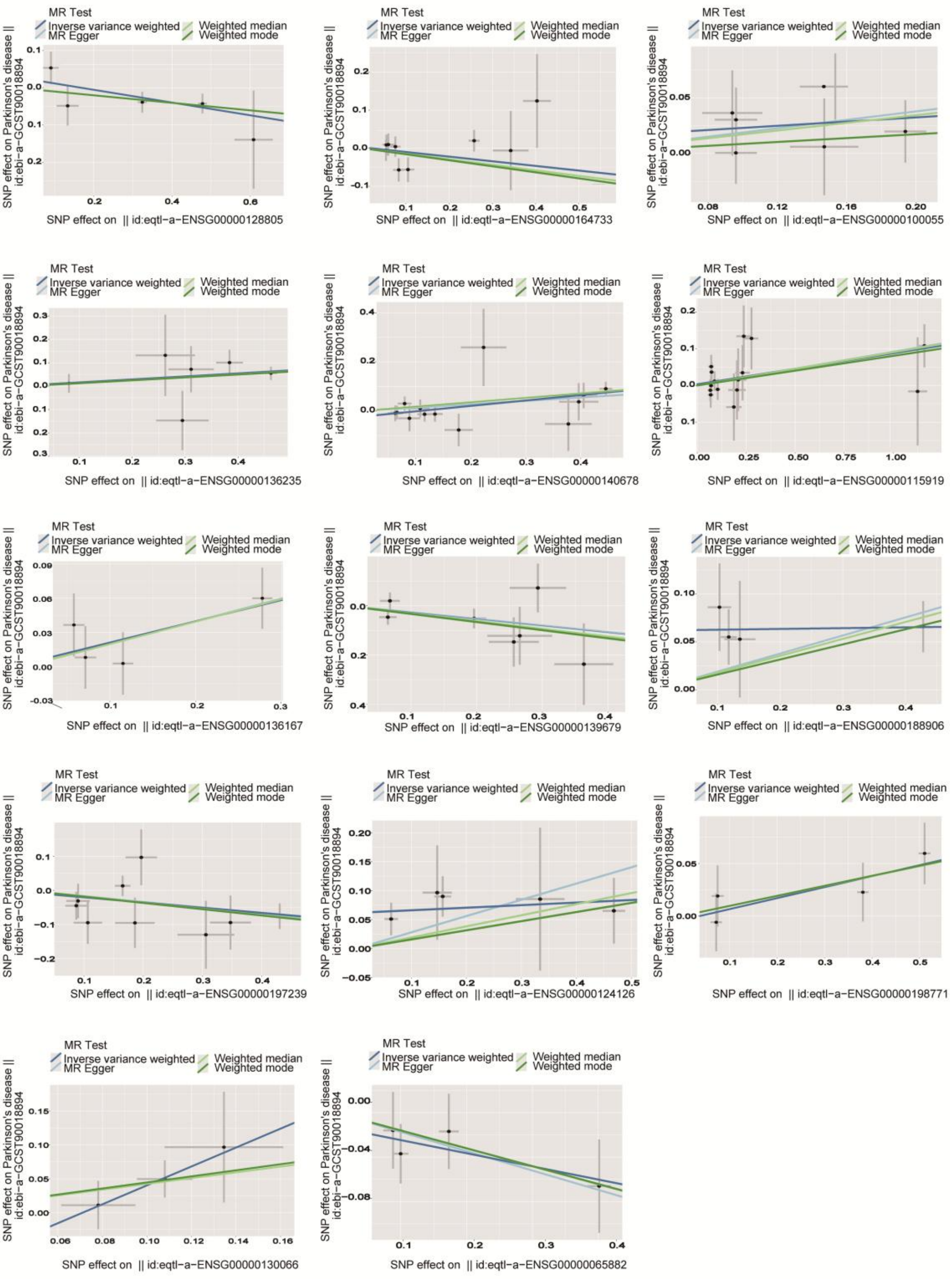

MR Test
Inverse variance weighted
MR Egger
Weighted median
Weighted mode
SNP effect on Parkinson's disease || id:ebi-a-GCST90018894
SNP effect on || id:eqtl-a-ENSG00000128805
SNP effect on || id:eqtl-a-ENSG00000164733
SNP effect on || id:eqtl-a-ENSG00000100055
SNP effect on || id:eqtl-a-ENSG00000136235
SNP effect on || id:eqtl-a-ENSG00000140678
SNP effect on || id:eqtl-a-ENSG00000115919
SNP effect on || id:eqtl-a-ENSG00000136167
SNP effect on || id:eqtl-a-ENSG00000139679
SNP effect on || id:eqtl-a-ENSG00000188906
SNP effect on || id:eqtl-a-ENSG00000197239
SNP effect on || id:eqtl-a-ENSG00000124126
SNP effect on || id:eqtl-a-ENSG00000198771
SNP effect on || id:eqtl-a-ENSG00000130066
SNP effect on || id:eqtl-a-ENSG00000065882

B

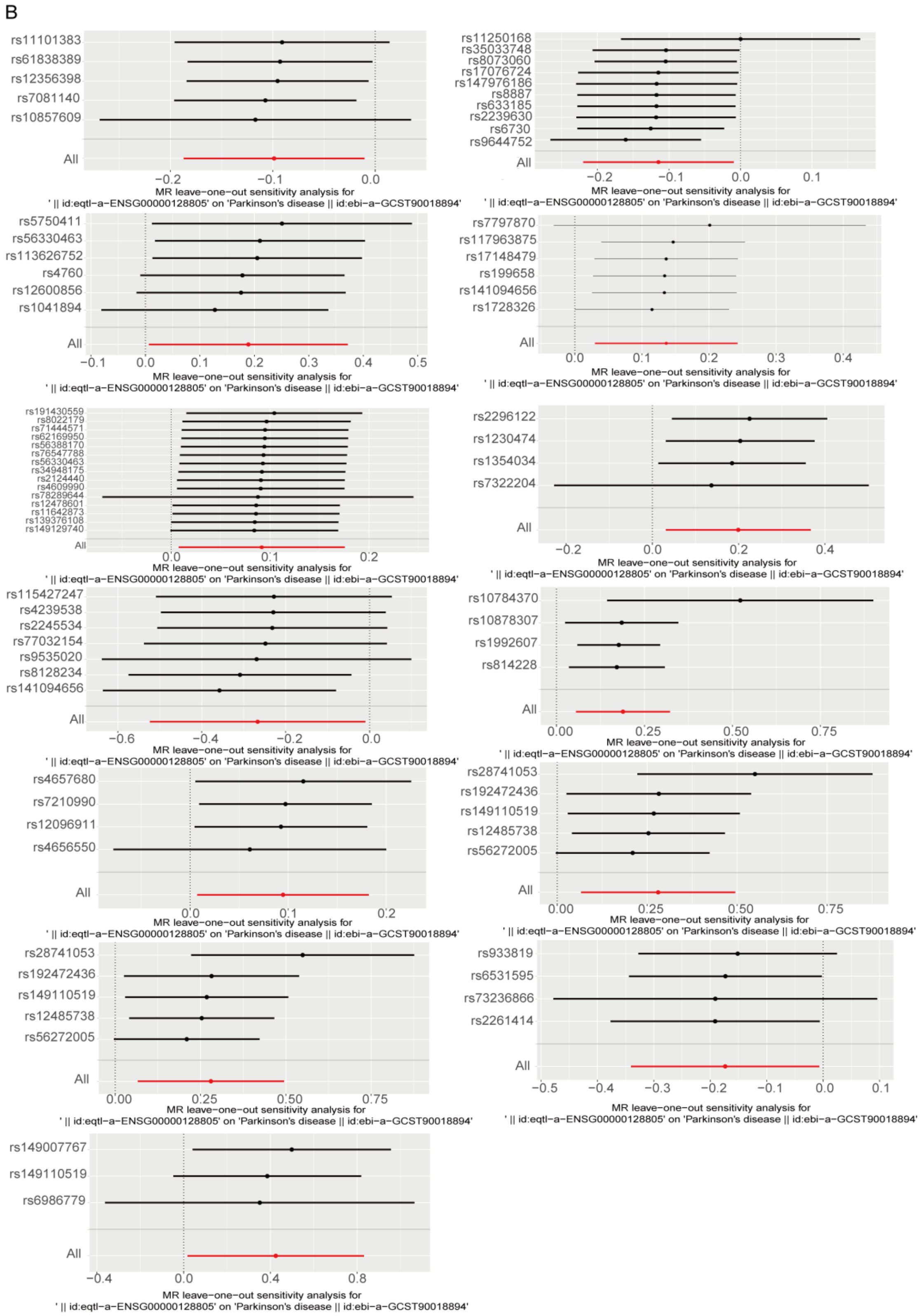

Supplementary Figure S2 Two-sample MR evaluating associations between PD risk and genetically predicted expression of candidate genes. A MR scatter plot showing SNP effect sizes on gene expression exposure against SNP effect sizes on PD risk outcome. Fitted lines for IVW, weighted median, weighted mode, and MR-

Egger models are displayed. **B** Leave-one-out sensitivity analysis forest plots. Each row represents an MR effect estimate omitting one instrumental-variable SNP; the "All" row denotes the overall incorporating all IVs, testing for bias driven by a single outlier instrumental variable.

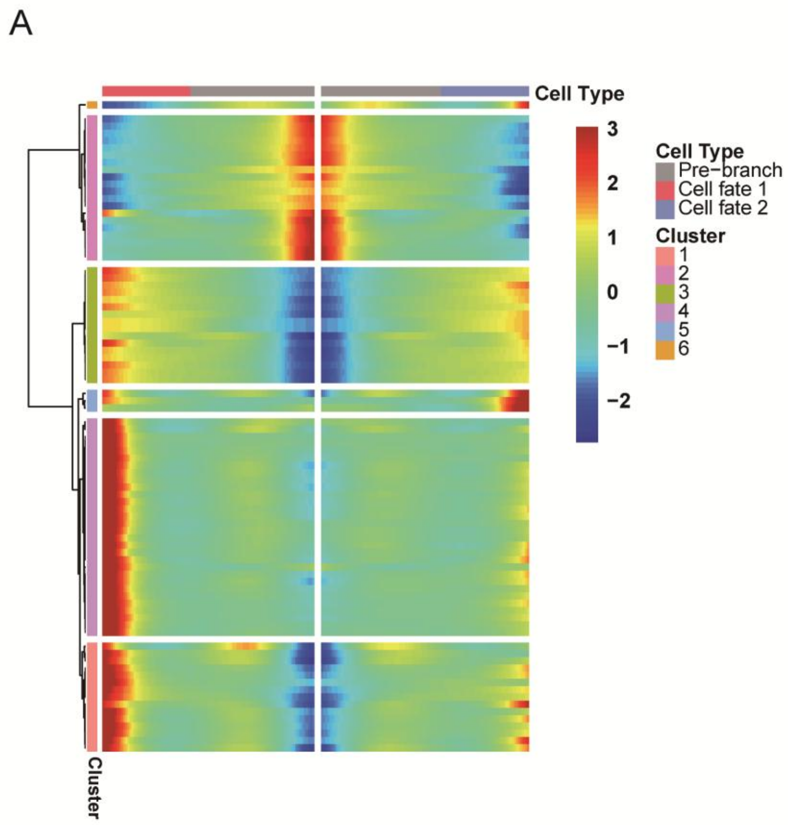

Supplementary Figure S3 Single-cell expression and pseudotime trajectory analysis of the three prioritized PD candidate genes. A heatmap illustrates the transcriptional dynamics of genes that show significant expression changes across different pseudotime trajectory branches. Each column represents an individual cell, while each row corresponds to a single gene.

Table S1

Table S1. Number of PVM nuclei retained per donor after quality control, used as the donor-level biological replicates in the pseudobulk differential expression analysis.

Donor	Group	Number of PVM nuclei
C1	Control	177
C2	Control	201
C3	Control	104
C4	Control	316
C5	Control	145
C6	Control	228
PD1	Disease	205
PD2	Disease	650
PD3	Disease	550
PD4	Disease	128
PD5	Disease	1116

Table S2

Table S2. Inverse-variance weighted (IVW) two-sample Mendelian randomization results for 18 cis-eQTL instruments prioritized from PVM differential expression. β and 95% CI are given on the log-odds scale; OR = exp(β). All 18 associations are treated as exploratory candidates and further ranked by sensitivity analyses and colocalization.

Gene	nSNP	β (95% CI)	P	OR (95% CI)
ARHGAP22	5	-0.099 (-0.187, -0.011)	0.028	0.906 (0.829, 0.989)
CTSB	10	-0.115 (-0.220, -0.010)	0.033	0.891 (0.803, 0.990)
CYTH4	6	+0.189 (0.007, 0.372)	0.042	1.208 (1.007, 1.451)
GPNMB	6	+0.136 (0.030, 0.243)	0.012	1.146 (1.030, 1.275)
HLA-DRA	5	-0.316 (-0.617, -0.016)	0.039	0.729 (0.540, 0.984)
ITGAX	13	+0.139 (0.043, 0.234)	0.005	1.149 (1.044, 1.264)
KYNU	15	+0.092 (0.008, 0.176)	0.033	1.096 (1.008, 1.192)
LCP1	4	+0.199 (0.031, 0.367)	0.020	1.220 (1.031, 1.443)
LPAR6	7	-0.267 (-0.524, -0.010)	0.042	0.766 (0.592, 0.990)
LRRK2	4	+0.189 (0.055, 0.323)	0.006	1.208 (1.057, 1.381)
OSBPL3	4	+0.144 (0.006, 0.282)	0.040	1.155 (1.006, 1.326)
PELI1	9	-0.171 (-0.307, -0.034)	0.014	0.843 (0.736, 0.967)
PREX1	5	+0.281 (0.066, 0.495)	0.010	1.324 (1.068, 1.640)
RCSD1	4	+0.095 (0.007, 0.182)	0.034	1.100 (1.007, 1.200)
RIN3	6	+0.200 (0.038, 0.362)	0.016	1.221 (1.039, 1.436)
SAT1	3	+0.425 (0.018, 0.832)	0.041	1.530 (1.018, 2.298)
TBC1D1	4	-0.174 (-0.340, -0.007)	0.041	0.840 (0.712, 0.993)
USP15	7	+0.127 (0.011, 0.242)	0.031	1.135 (1.011, 1.274)

Table S3

Table S3. Colocalization statistics for the four prioritized loci, using default coloc priors (p1 = p2 = 1×10^{-4}, p12 = 1×10^{-5}). PP.H4 is the posterior probability that the eQTL and PD GWAS signals share the same causal variant at the lead SNP. PP.H3 and the full variant count within each region are to be completed against the R coloc summary output.

Gene	Lead SNP	Variants analysed	PP.H3	PP.H4
HLA-DRA	rs9273500	1	—	0.958
OSBPL3	rs34105255	1	—	0.997
RIN3	rs17783630	1	—	1.000
USP15	rs1920099	1	—	1.000

Table S4

Table S4. The primer sequences used for mouse genes.

Name	Forward	Reverse
Actb	CTGGCCTCACTGTCCACCTT	GGGCCGGACTCATCGTACT
H2-Ea	GAGGGACGATCACCTCTTOC	CCAGTGATCCACCTCACAGT
Osbpl3	ATGACTTACACCCAGGAGC	GAAGAATCTCTTGTGCCAGC
Rin3	GTCAGTAGAGACTTACTGCCC	GGTCTCAAGTTCAAGGAAGC
